# Emerging Technologies to Control Oocyte Apoptosis Are Finally Treading on Fertile Ground

**DOI:** 10.1100/tsw.2001.39

**Published:** 2001-05-01

**Authors:** Jonathan L. Tilly

**Affiliations:** Vincent Center for Reproductive Biology, Department of Obstetrics and Gynecology, Massachusetts General Hospital/Harvard Medical School, Boston 02114, USA

**Keywords:** infertility, menopause, apoptosis, cell death, oocyte, ovary, sphingosine-1-phosphate

## Abstract

As the medical community strives to improve on the efficacy of anticancer treatments, a critical issue not to be overlooked since the overall quantity of life has been substantially increased in many cancer survivors is the quality of that life post-therapy. Indeed, one of the most worrisome side effects of conventional cancer treatments is damage to the gonads. This problem is compounded in females since the ovaries, unlike the testes, are incapable of germ cell renewal in postnatal life. As a consequence, the inappropriate destruction of female germ cells (oocytes) following exposure to chemotherapeutic drugs and radiation is irreparable, often leading to premature menopause and infertility [1]. Considering recent estimates that 1 in 52 human females between birth and age 39 (i.e., the pre-reproductive and reproductive years) will be diagnosed with, and presumably treated for, cancer [2], new strategies to minimize or prevent gonadal damage during such treatments would have a profound positive impact on millions of lives.

In this regard, a recent study published in *Nature Medicine* has offered some hope that technologies aimed at preserving ovarian function and fertility in female cancer patients are indeed on the horizon [3]. Using mice as the model system, it was reported that pretreatment of young adult females with a single dose of a naturally occurring lipid referred to as sphingosine-1-phosphate (S1P) prior to irradiation completely protected the ovarian germ cell population from this pathological insult. By comparison, females pretreated with vehicle exhibited massive oocyte depletion, a characteristic ovarian response to radiotherapy. Why was S1P so effective? In the same report, genetic evidence was provided implicating the pro-apoptotic molecule, ceramide [4], as the principal messenger responsible for relaying the insult to the core cell death machinery in oocytes [3]. Given that S1P has been documented to function as a potent antagonist of cellular stress responses and apoptosis induced by ceramide [5], the protection afforded to oocytes by S1P is thus probably a reflection of this biological activity as well as of the importance of ceramide to oocyte death.

Over the years, a number of investigators have attempted to devise methods that hold promise for preserving fertility, if not ovarian function, in female cancer patients [6]. Unfortunately, the vast majority of approaches have thus far met with little success, probably due, at least in part, to a lack of understanding of the basic mechanisms responsible for oocyte depletion observed in these patients. To fill in this gap, a study published in *Nature Medicine* in 1997 indicated that oocytes exposed to the common chemotherapeutic drug, doxorubicin, did not simply wither away in some nonspecific or pathological manner but rather initiated the programmed cell death process of apoptosis [7]. This piece of information turned out to be invaluable since one could then hypothesize that controlled manipulation of key components of the oocyte death program represents a novel approach to "shield" the ovaries from the ravages of anticancer therapies. Indeed, it was further shown that a targeted disruption of the gene encoding Bax, a pro-apoptotic member of the *bcl-2* gene family [8], protected oocytes *in vivo* from repeated doxorubicin treatment. Despite these insights into how anticancer therapies damage the ovaries, clinical application of these findings remained purely speculative since one cannot simply "knock out" the *bax* gene (or any other gene, for that matter) in humans, hence the obvious potential values of a small molecule like S1P for clinical development.

The approach taken by Morita et al. [3] was to inject vehicle (controls) or S1P into the bursal cavity surrounding each ovary 2 hr prior to administration of a dose of ionizing radiation sufficient to kill off roughly 80% of the resting (primordial) oocyte population. In this way, delivery of S1P was confined to the ovaries, thus precluding the possibility of unwanted protective effects of S1P in the cancer cells that might have arisen if the substance was provided via systemic routes. At 2 weeks post-irradiation, the ovaries were collected and processed for two endpoints: oocyte numbers and the developmental potential of the oocytes following fertilization. For both endpoints, no differences were observed between mice that had not been irradiated vs. those protected by S1P *in vivo* prior to irradiation. These findings starkly contrasted the pronounced oocyte loss and markedly reduced developmental potential of the oocytes that did remain in the ovaries of irradiated females pretreated with vehicle. Furthermore, preliminary results from *in vivo* mating trials discussed in this publication [3] support the conclusion that S1P indeed preserves fertility in female mice exposed to anticancer therapy.

So, the burning question that remains is "what's next?" First, experiments are needed to rigorously test the quality of the "protected" oocytes. In this regard, several approaches, ranging from assessments of aneuploidy rates to germ line transmission of potential genetic mutations to offspring conceived from the "protected" oocytes, are currently underway. Until the final results from these studies are available, we remain encouraged by reports that children conceived by mothers treated previously for cancer show no increase in the incidence of congenital malformations when compared with offspring of nontreated siblings [9]. Second, the relevance of data derived from studies of mouse ovaries to human ovaries needs to be established. Since anticancer therapies preferentially destroy immature oocytes, use of "spare" human oocytes from assisted reproductive technology programs may be of little value since these germ cells are generally collected at mature stages of development. In addition, these experiments would, of course, suffer from all of the *in vitro* caveats one could imagine. Nevertheless, through a collaboration with Dr. R.F. Casper (Mount Sinai Hospital, Toronto, Ontario, Canada), we feel that hope still exists for pre-clinical testing of such strategies in human oocytes at the appropriate stage of development, *in vivo*, by employing mice harboring grafted human ovarian cortical tissue fragments as a model system [10]. While results from these ongoing experiments are still too early to discuss in detail, answers to all of these important questions will hopefully be provided in the near future. Lastly, the potential use of S1P and other as yet unreported "germ cell preserving strategies" to slow the normal depletion of oocytes leading to menopause in women should not be overlooked. For example, in mice it has been established that Bax is required for oocyte apoptosis induced by both developmental cues and anticancer therapy [7,11]. If a similar conservation of the ceramide/S1P rheostat exists in these two distinct paradigms of oocyte death, it is conceivable that S1P therapy could prolong normal ovarian function in a manner similar to that recently achieved, at least in female mice, by *bax* gene knockout [11]. However, the possibility of postponing menopause, with all of its biological and ethical ramifications, is perhaps best left for another day.

## References

1. Morita, Y. and Tilly, J.L. (1999) Oocyte apoptosis: like sand through an hourglass. *Dev. Biol.* **213,** 1–17.
2. Greenlee, R.T., Murray, T., Bolden, S., and Wingo, P.A. (2000) Cancer statistics, 2000. *CA – A Cancer Journal for Clinicians* (American Cancer Society) **50,** 7–33.
3. Morita, Y., Perez, G.I., Paris, F., Miranda, S., Ehleiter, D., Haimovitz-Friedman, A., Fuks, Z., Xie, Z., Reed, J.C., Schuchman, E.H., Kolesnick, R.N., and Tilly, J.L. (2000) Oocyte apoptosis is suppressed by disruption of the *acid sphingomyelinase* gene or by sphingosine-1-phosphate therapy. *Nat. Med.* **6,** 1109–1114.
4. Kolesnick, R.N. and Kronke, M. (1998) Regulation of ceramide production and apoptosis. *Annu. Rev. Physiol.* **60,** 643–665.

5. Spiegel, S. and Milstien, S. (2000) Sphingosine-1-phosphate: signaling inside and out. *FEBS Lett.* **476,** 55–57.
6. Blumenfeld, Z., Avivi, I., Ritter, M., and Rowe, J.M. (1999) Preservation of fertility and ovarian function and minimizing chemotherapy-induced gonadotoxicity in young women. *J. Soc. Gyn. Inv.* **6,** 229–239.
7. Perez, G.I., Knudson, C.M., Leykin, L., Korsmeyer, S.J., and Tilly, J.L. (1997) Apoptosis-associated signaling pathways are required for chemotherapy-mediated female germ cell destruction. *Nat. Med.* **3,** 1228–1232.
8. Antonsson, B. and Martinou, J.C. (2000) The Bcl-2 protein family. *Exp. Cell. Res.* **256,** 50–57.
9. Lantz, M.E. (1999) In *Cancer Obstetrics and Gynecology*. Trimble, E.L. and Trimble, C.L., Eds. Lippincott Williams & Wilkins, Philadelphia, pp. 87–98.
10. Weissman, A., Gotlieb, L., Colgan, T., Jurisicova, A., Greenblatt, E.M., and Casper, R.F. (1999) Preliminary experience with subcutaneous human ovarian cortex transplantation in the NOD-SCID mouse. *Biol. Reprod.* **60**, 1462–1467 (1999).
11. Perez, G.I., Robles, R., Knudson, C.M., Flaws, J.A., Korsmeyer, S.J., and Tilly, J.L. (1999) Prolongation of ovarian lifespan into advanced chronological age by *Bax*-deficiency. *Nat. Genet.* **21,** 200–203.

Received: February 9, 2001
Accepted: March 26, 2001
Published: May 1, 2001
